# Brain tissue oxygen tension: Is it a derivative of arterial blood?

**DOI:** 10.1186/s13054-022-04130-w

**Published:** 2022-09-23

**Authors:** Gurgen Harutyunyan, Varsenik Harutyunyan Jaghatspanyan, Emma Martirosyan, Rosa Isabel Benitez Bermejo, Garnik Harutyunyan, Andrés Sánchez Gimeno, Pau Ignasi García Zapata, Armen Varosyan, Suren Soghomonyan

**Affiliations:** 1 Emergency Department, Hospital 9 de Octubre, VITHAS, Valle de la Ballestera 59, 46015 Valencia, Spain; 2 Faculty of Pharmacy, Universitat De València, C. del Cementerio, 1, 46100 Burjassot, Valencia Spain; 3 Faculty of General Medicine, Yerevan State Medical University, 2 Koryun St, 0025 Yerevan, Armenia; 4 Consorcio Hospital General Universitario de Valencia, Av. de les Tres Creus, 2, 46014 Valencia, Spain; 5 Erebouni Medical Center, Titogradyan St. 14, 0087 Yerevan, Armenia; 6 Clinical Department of Anesthesiology, The Ohio State University Wexner Medical Center, N411 Doan Hall, 410 West 10th Avenue, Columbus, OH 43210 USA

## Introduction

The article of Thomas Gargadennec’s et al. “Detection of cerebral hypoperfusion with a dynamic hyperoxia test using brain oxygenation pressure monitoring” [1] is a big step forward towards a new paradigm in neurotrauma: the high brain tissue oxygen pressure (PbrO_2_) presence by oxygen challenge (OC) from baseline to 100% in brain-injured patients is in fact independent from local perfusion sufficiency (i.e. the cut-off of regional cerebral blood flow < 3.5 ml/100 gxmin). Accordingly, with OC the PbrO_2_ in the tissue of traumatic brain injury (TBI) patients without hypoperfusion reaches up to 123 [96–138] mmHg (supplement 2) [1].

This daily challenge of PbrO_2_, whose mechanisms of action in the end capillaries remain uncertain until today, is explained by authors as an “increase in interstitial oxygen diffusion at the arterial capillary side” [1].

Indeed, with OC in all groups of traumatic and non-traumatic brain injury patients, the PbrO_2_ reaches to arterial oxygen pressure (PO_2_) levels (i.e. 62 mmHg in hypoperfusion zones and 91 mmHg in no brain hypoperfusion zones). Therefore, the blood that is in said environment has to be arterial.

On the other hand, as confirmed by Johnston and colleagues, “normally it is assumed that there is a minimal oxygen gradient between the extracellular space and the end-capillary compartment, and thus that PbrO_2_ reflects end-capillary oxygen tension” [2].

As we know, the Clark electrode measures PO_2_ in a volume of 1 mm^3^, where there are millions of cells and hundreds of capillaries; this “small” volume encloses such a “megacontent” which is practically in an environment of the same pressure. Consequently, the end-capillary PO_2_ in this volume is at least equal or higher than the PO_2_ measured by PbrO_2_ electrode.

Accepting data presented in the article that the changes of PbrO_2_ by OC in all brain-injured patients raise to arterial levels of PO_2_, we can confirm that in a fairly large homogeneous brain volume, the venous capillary side blood has arterial level of PO_2_ by hyperoxia. As confirmation, the MRI-derived brain extracellular PO_2_ data with OC (which includes a much larger volume of tissue) are consistent with data from the literature obtained using invasive techniques and exceed 100 mmHg [3].

However, current literature indicates no significant change in cerebral metabolic rate of brain tissue oxygen consumption by normobaric hyperoxia [4–7] and oxygen extraction fraction (OEF) at 0.56 ± 0.06 in reversible tissues [8]. That is, the OC at the end of cerebral capillaries causes high PO_2_ which is typical to arterial blood with the presence of blood with low oxygen saturation of Hb (SO_2_) (i.e. venous blood).

With the classical knowledge, it is impossible to explain the presence of such a high PO_2_ at the end-capillary side of brain tissue: according to the sigmoid “S”-shaped oxyhaemoglobin dissociation curve (ODC), the SO_2_ with OC in the brain tissue end-capillary part is expected to be near 100%, which would mean the miserly oxygen extraction and massive mitochondrial dysfunction by hyperoxia.

The solution of this puzzle is in the field of biochemistry: the described high increase in PbrO_2_ with OC is possible only with intracapillary conformational change of haemoglobin (Hb) quaternary state from relaxed (R) to tens (T), which has a lower Hb–O_2_ affinity, highest buffering capacity and hyperbolic and low form of ODC [9].

The existence of Hb T state in the cerebral microcirculation is essential: first, it increases PO_2_ with low SO_2_ in the capillary venous part. Second, it favours to equally distribute PO_2_ among all cells by capillary length in homogeneous tissue. And finally, it incomparably increases Hb buffering capacity to maximum, reaching the human Haldane coefficient at 0.6 (i.e. the release of 1 mol of oxygen will allow the Hb to bind a 0.6 mol of H +) [9].

Assuming this, we can confirm that the increase in PbrO_2_ by OC is a phenomenon due to T state of Hb in the cerebral venous capillary side with or without local perfusion involvement. Furthermore, the biological sense of cerebral autoregulation is to maintain Hb T quaternary state in the cerebral end-capillary part.

Acknowledgements are due to the authors who confirm the presence of arterial PO_2_ equivalent PbrO_2_ with OC in various types of brain injury patients, regardless of the state of local perfusion.

Thanks to this practical discovery and the biochemical explanation of the process (i.e. intracapillary R to T transition of Hb), many discrepancies in neurotrauma patients can be clarified (we have discussed in detail elsewhere) [10, 11].

## Conclusion

Brain tissue oxygen pressure is derived from end-capillary oxygen tension independent of oxygen challenge and reflects the T state of haemoglobin.

## Data Availability

Not applicable.
